# “It's a lot more complicated than it seems”: physiotherapists' experiences of using compensation strategies in people with Parkinson's

**DOI:** 10.3389/fresc.2023.1157253

**Published:** 2023-06-05

**Authors:** Sheemah Alenezi, Sarah Morgan-Trimmer, Sophia Hulbert, William Young, Victoria A. Goodwin

**Affiliations:** ^1^ College of Applied Medical Sciences, Physical Therapy and Rehabilitation Department, Jouf University, Al-Qurayyat, Saudi Arabia; ^2^ Faculty of Health and Life Sciences, Exeter University, Exeter, United Kingdom; ^3^ School of Health Professions, Faculty of Health, Plymouth, United Kingdom

**Keywords:** compensation strategies, cueing, external cues, internal cues, person-centered care, Parkinson’s disease

## Abstract

**Background:**

Gait disturbances often result in functional limitations in daily activities and negatively impact the quality of life in people with Parkinson's disease. Physiotherapists often employ compensation strategies in an attempt to improve patients' walking. However, little is known about physiotherapists' experiences in this regard. We evaluated how physiotherapists adopt compensation strategies and what they draw on to inform their clinical decision-making.

**Methods:**

We carried out semi-structured online interviews with 13 physiotherapists with current or recent experience working with people with Parkinson's disease in the United Kingdom. Interviews were digitally recorded and transcribed verbatim. Thematic analysis was utilized.

**Results:**

Two main themes were developed from the data. The first theme, optimizing compensation strategies through personalized care, shows how physiotherapists accounted for the individual needs and characteristics of people with Parkinson's, which resulted in them individually tailoring compensation strategies. The second theme, delivering compensation strategies effectively, considers the available support and perceived challenges with work settings and experience that impact physiotherapists' ability to deliver compensation strategies.

**Discussion:**

Although physiotherapists strived to optimize compensation strategies, there was a lack of formal training in this area, and their knowledge was primarily acquired from peers. Furthermore, a lack of specific knowledge on Parkinson's can impact physiotherapists’ confidence in maintaining person-centered rehabilitation. However, the question that remains to be answered is what accessible training could address the knowledge–practice gap to contribute to the delivery of better-personalized care for people with Parkinson's.

## Introduction

Gait disturbances are among the most debilitating motor symptoms of Parkinson's disease, they result in functional limitations of daily activities and negatively impact the quality of life (1). People with Parkinson's (PwP) often experience gait disturbances, including but not limited to reduced gait speed and step length (1) and freezing of gait (2) that are associated with falling (3). Dopaminergic treatments are primarily effective in managing motor symptoms; however, gait disturbances are often resistant to pharmacological treatment (4). Rehabilitation interventions, such as treadmill usage and strategy training (5), have been shown to improve gait in PwP.

Compensation strategies are key to managing gait disturbances in PwP (6); they are described as “complex motor sequences and cueing interventions that use temporal or spatial external stimuli associated with the initiation and ongoing facilitation of motor activity” (5). These compensation strategies include cueing (7), motor imagery and action observation (6). Furthermore, dual-task training (8) has been searched extensively; however, it might not typically be considered a compensatory strategy. The evidence of effectiveness of these strategies is mixed. For example, dual-task training is considered one of the most common strategies (9). However, a recent review of physiotherapy intervention for Parkinson's disease showed that this intervention is not effective in improving gait outcomes (i.e., gait speed, step length, and freezing of gait) in the short term (5). Meanwhile, action observation and motor imagery have been shown to be effective in managing gait disturbance in PwP (10).

A growing body of literature recognizes that compensation strategies work differently in every person with Parkinson's (6). The literature also emphasizes the necessity of personalized rehabilitation for gait disturbances in PwP (11). However, what is not yet understood is how physiotherapists use these strategies in their everyday, routine practices and what factors may influence their choices regarding Parkinson's rehabilitation.

Tosserams et al. (12) conducted a survey and found that physiotherapists lacked awareness of compensation strategies other than cueing. In another study, the authors reported the difficulty of integrating compensatory strategy training into clinical practice for PwP (13). A scoping review of the use of implicit movement strategies in the management of gait impairments in PwP found no studies that explored the perspectives of physiotherapists regarding the use of various strategies (14). This highlights the need to understand better physiotherapists' experience in recommending strategies and the knowledge that they draw upon to inform this clinical decision-making. Therefore, this study aims to explore physiotherapists’ understanding and experiences of using compensation strategies for PwP.

## Methods and materials

### Research design

This study used a qualitative approach, to provide in-depth insights into the real-world experience of people (15). Interviews were facilitated using a semi-structured topic guide (Supplementary File) which focused on how physiotherapists learn about compensation strategies to understand what they draw on for their clinical practice. Interviewees were also asked their opinions on the effectiveness of compensation strategies and how they use them to understand their approach to optimizing compensation strategies.

The topic guide was piloted with a physiotherapist to ensure the clarity of interview questions. This study reported according to the 32 items of Consolidated Criteria for Reporting Qualitative Research checklist (16).

### Ethics

This study was approved by the Ethics Committee of the College of Medicine and Health Research of Exeter University (Reference: Mar21/B/266 on 02/03/2021).

### Participants and recruitment

A purposive sampling was carried out of United Kingdom (UK)-based physiotherapists with current or recent experience working with PwP. To determine eligible physiotherapists, it was important to identify physiotherapists who have experience working with PwP. The UK government's National Institute for Health and Care Excellence guidelines suggest that physiotherapists who specialize in Parkinson's disease should treat PwP (17). However, there are relatively few such specialists in the UK, and many PwP are seen by physiotherapists with broader ranges of expertise, including those who work with older people or who have patients with other neurological conditions. Some of the members of the latter group may work in non-National Health Services (NHS) settings, such as in the private, charitable, or higher education sectors. Thus, physiotherapists who considered themselves specialists in Parkinson's or who had experience working with PwP within a broader practice were deemed eligible. Participants were purposively selected to ensure maximum variation in their characteristics [i.e., gender, work settings (community, in-patient, and out-patient), geographical location, and level of expertise in managing Parkinson's].

Physiotherapists were recruited via national networks such as the Parkinson's Excellence Network and AGILE (Chartered physiotherapists working with older people), which offered support in identifying potential participants. Social media platforms can promptly capture larger numbers of targeted participants (18); therefore, Twitter and Facebook were used to promote the study, with key organizations and individuals tagged to facilitate snowball recruitment. Interested participants were provided with an information sheet explaining the study's aim and the reasons for various selection criteria. Written informed consent was obtained from participants prior to commencing interviews.

### Data collection

Interviews were conducted by SA, a female physiotherapist by background and PhD candidate who had no prior relationship with any participants. She had undertaken training in qualitative research methods as part of her PhD but was new to conducting this type of research. All interviews were conducted on the Microsoft Teams platform due to the social distancing required by the COVID-19 pandemic. The interview periods ranged from 36 to 75 min. At the end of each interview, participants had the opportunity to elaborate on any aspect that may not have been discussed in detail. Interviews were digitally recorded and transcribed verbatim, with transcriptions completed by an external transcriber. The interviewer then checked the transcripts against the recordings to ensure their accuracy. The transcripts were anonymized and retained securely. The data were collected between March and October 2021.

Regarding the sample size of the interview studies, the model of information power over data saturation has been adopted (19). This study used rich interview data from 13 physiotherapists with different ranges of Parkinson's expertise and work settings around the UK to answer the research questions.

### Data analysis

Data were analyzed using thematic analysis, enabling an in-depth description of patterns of meaning in the collected data (20–22). The analysis adopted a critical realist perspective (23) to explore participants' experience using compensation strategies (realist ontology) while acknowledging the variation between participants (relativist epistemology). Six phases of thematic analysis were conducted iteratively (Table 1).

**Table 1:** Phases of the thematic analysis.

Phases of thematic analysis	Building the trustworthiness
Phase one: Familiarizing yourself with your data	The first author read and re-read the transcripts, wrote down early thoughts and ideas relevant to research questions, and made notes about the most frequent/recurrent topics reported by participants. For example: - Participants considered using compensation strategies as an ongoing process. - Participants were aware of contextual factors that impact how strategies work. - Participants emphasized the need for support from family and rehabilitation team members.
Phase two: Generating initial codes	Given that there is little theory and knowledge in the literature to draw on, inductive coding was prioritized to synthesize key research questions (i.e., how physiotherapists use compensation strategies). Two authors (SA and VG) independently coded two transcripts and compared them to enhance data interpretation before SA coded all transcripts. Later, SA discussed developed codes with the rest of the team (SM-T, SH, WY and VG) to enhance the systematic coding process.
Phase three: Searching for themes	The developed codes were grouped into categories representing initially three themes around personalizing compensation strategies, facilitators, and barriers preventing physiotherapists from delivering these strategies (Table 2).
Phase four: Reviewing themes	Themes were utilized as defined as “central organizing concept (22: p.9).” While checking the data against the initial theme of personalizing compensation strategies, it has been generated the idea that a physiotherapist’s understanding of a PwP's needs (physical and psychological) and how these needs change over time (due to the disease) not only lead them to tailor their approach to meeting these needs, but also viewing these considerations was dynamic that needs constant reviewing. They were also building a relationship with PwP to raise their awareness of education, leading to bigger impacts and meaningful engagement to enhance PwP motivation. In addition, physiotherapists enabled PwP to use strategies and consider the support they need, for example, involving a partner. These sub-themes were centered on physiotherapists’ approach to optimizing compensation strategies (Figure 1). Furthermore, many barriers and facilitators for this treatment were the product of specific work settings. By understanding variations within workplaces (hospital-based and community-based) and how available support differ within work settings (including the rehabilitation team member) was different, it has been conceptualized that physiotherapists’ service deliveries were shaped by their relationship to the work setting as well as their prior knowledge and experience (Figure 2). These themes ended up grouping two original themes into one theme centered on factors that impacted physiotherapists’ effective delivery of movement strategies. These themes were refined and reviewed with the whole team in group meetings.
Phase five: Defining and naming themes	To ensure that each theme named captured the meaning of any included subthemes.
Phase six: Producing the report	Writing up the findings and sharing them with the group team.

**Figure 1:**
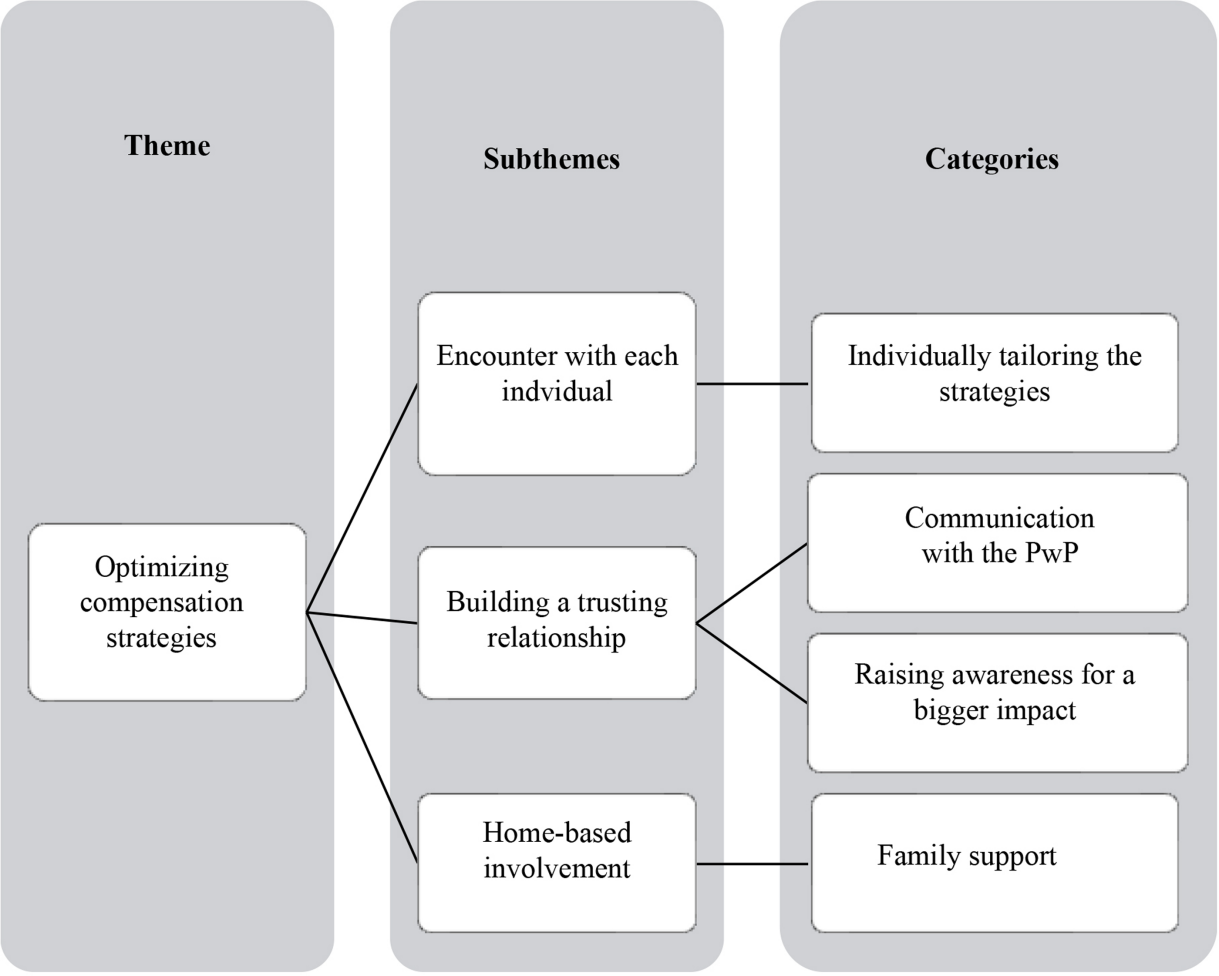
How physiotherapists optimize compensation strategies.

**Figure 2:**
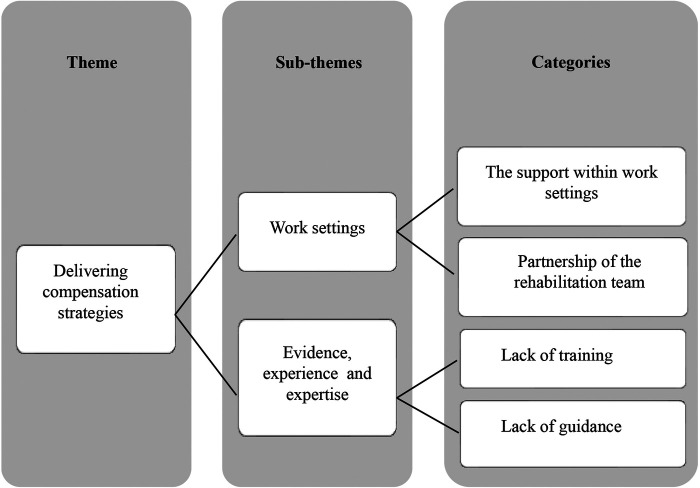
How physiotherapist deliver compensation strategies.

Many considerations were taken to establish the trustworthiness of thematic analysis (24). First, two researchers were involved in the coding process to ensure triangulation. In addition, the systematic coding process was enhanced through regular discussions of team meetings. Second, the audited trials (Table 2) provide clarity of synthesis and explanation of the process of developing themes. Thirdly, the study findings were discussed through team meetings and with participant's feedback and thoughts to help us improve clarity.

**Table 2:** Example of audited trail.

Codes	Categories	Initial themes
PwP’ characteristics • Age • Disease severity • Cognition • Musculoskeletal problems • Comorbidities • Psychological needs	Individually tailoring strategies	Personalizing compensation strategies
PwP’ preferences • Routines • Beliefs • Previous experiences • Understanding	Valuing patient needs and preferences	
• Engagement • Motivation	Communication with PwP	
• Education	Raising patient awareness for a bigger impact	
Family role • Help • Supervision • Provisions of reminders	The support	Facilitators
• Access to the rehabilitation team	The support	
• Lack of guidance • Lack of training	The challenges	Barriers

## Results

### Participants

Twelve of the 13 interviewees were female, and participants had three to thirty years of clinical experience (Table 3). The participants were physiotherapists who identified themselves as Parkinson's specialists, physiotherapists who work in neurorehabilitation with half of their caseload being PwP, and physiotherapists who work with older people. The geographical location was not reported to ensure anonymity, as participant numbers are small. However, all participants practiced physiotherapy in the UK.

**Table 3:** Participant characteristics.

Participant characteristics	*N*
Gender	12 female 1 Male
Specialty	Parkinson's disease (3) Neurorehabilitation (5) Older people rehabilitation (5)
Work settings	Rehabilitation ward (7) Community-based (3) Community hospital (3)
Employment	NHS (9) Private (2) Academic (2)
Number of years of experience as a physiotherapist	3–10 (5) 11–20 (4) 20–30 (4)

**Figure 3:**
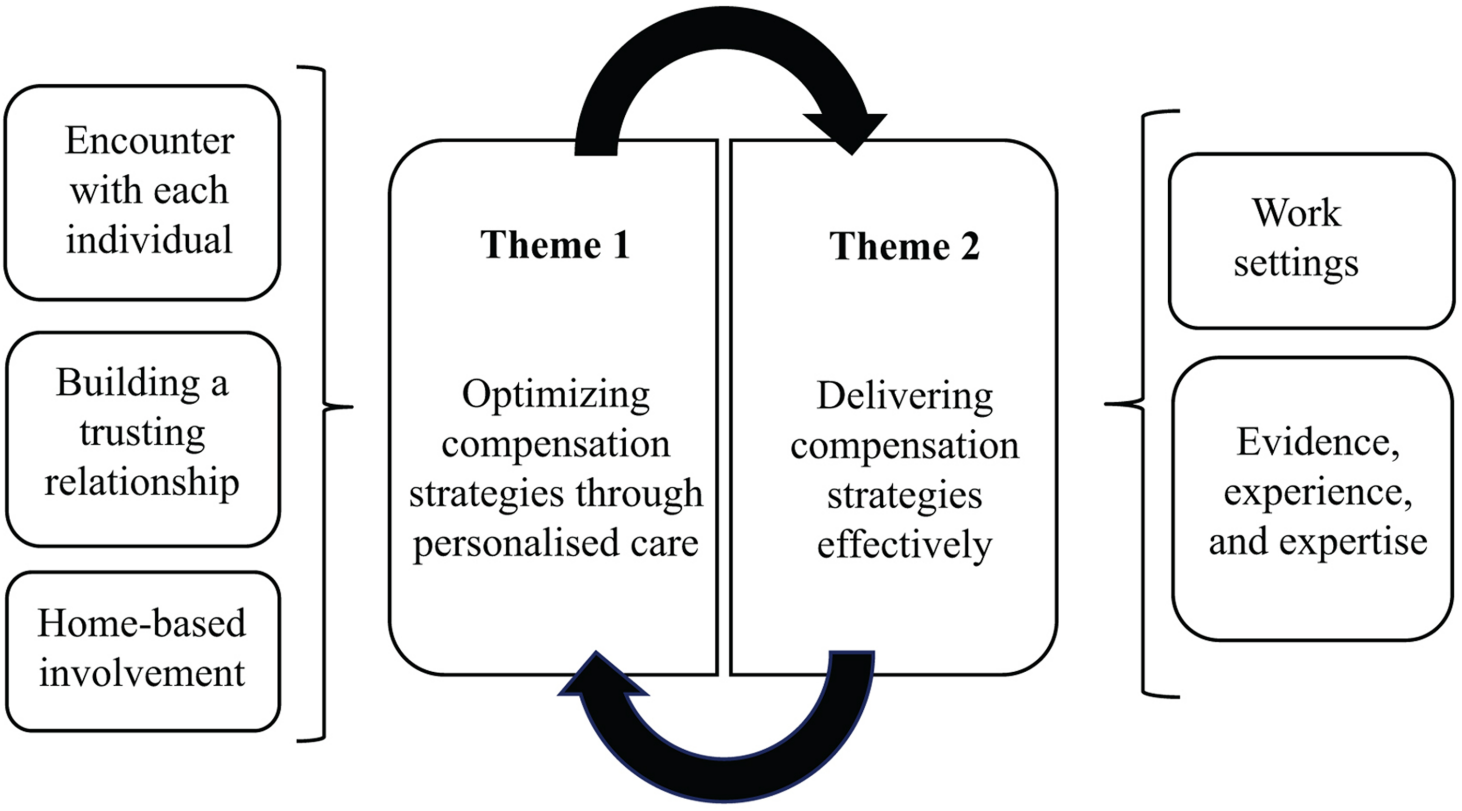
The final developed themes.

Two main themes were developed from the data. The first theme, optimizing compensation strategies through personalized care, relates to physiotherapists accounting for the individual needs and characteristics of PwP, resulting in them personalizing compensation strategies. The second theme, delivering compensation strategies effectively, can be ascribed to the available support and perceived challenges in work settings, which impact physiotherapists' ability to deliver compensation strategies (Figure 3).

#### Theme 1: optimizing compensation strategies through personalized care

Physiotherapists highlighted the use of compensation strategies to improve walking. However, this benefit depended on the individual characteristics of the PwP. Three subthemes were developed: (1) The encounter with each individual describes how physiotherapists encountered the diverse characteristics and needs of the PwP that led them individually to tailor compensation strategies. (2) Building a trusting relationship discusses the physiotherapists’ approach to engaging and motivating PwP to utilize compensation strategies. (3) Home-based involvement discusses the need for support to enable PwP to use compensation strategies on a daily basis in a home setting.

#### Subtheme 1: encounter with each individual

Understanding the individual characteristics of PwP was a guiding factor in the physiotherapists' approach to using compensation strategies. For example, physiotherapists reported that the person's cognition and level of disease severity were important considerations due to the potential impact on understanding and performing strategies. In addition to age, musculoskeletal problems and comorbidities were important because they influenced the person's responses. These considerations were viewed as dynamic because the characteristics of Parkinson's patients can change over longer periods of time. Therefore, a central part of personalization was ensuring that strategies specifically worked with the patient.

“Breaking down the cues—they’re internal, or external. So, they’re either going to be generated from within the person or external, like a visual one. So, obviously, if they’ve got a cognitive deficit, the internal ones are much harder. They’re better with an external one … whether it's auditory or visual. It's very much the individual thing, and you find what works for the patient. (Participant 5)”

The account of these needs was not limited to disease manifestations. A further consideration was addressing the psychosocial characteristics of the PwP. This was evident in the physiotherapists' understanding of the holistic approach to managing PwP.

“I have moved—when I started … I would be very much focusing on strength, balance, confidence, coordination. But I’ve moved so far from that to the other end of the scale that … I can measure their strength, and I can measure their balance, and it doesn’t really change. But their psychosocial bit tends to be my biggest block, and I’ve shifted my perspective away almost from being the physio to making sure that everything else is trying not to be out of kilter. (Participant 8)”

Understanding psychological aspects as a part of tailored or personalized care was evident in physiotherapists referring to other members of the multidisciplinary rehabilitation teams to optimize compensation. This is particularly the case of non-motor symptoms that can influence walking while they train compensation strategies.

“If there's a lot of anxiety around freezing, we might also—apart from doing the cues and strategies, the [occupational therapist (OT)] might work on some anxiety management. We also have a psychologist. So, we might work together with them as well—particularly if it's anxiety that's creating a vicious circle of freezing and not managing the movement because the anxiety's become too much and the strategies the OTs tried are not effective for various reasons. (Participant 5)”

Participants described how they used meaningful strategies by evaluating PwP' needs and preferences and tailoring strategies accordingly. For example, they ensured that strategies fit a person's interests (like their favorite music) or were previously used and familiar to them, making them easier or more comfortable to use.

“Is there a different sort of cue that you can give them that will make it less obvious to other people? Because they don’t want to feel self-conscious that people are watching them and thinking, that's a bit odd! (Participant 5)”

These interrelated considerations were viewed as dynamic, and optimizing these strategies was described as a constant process. Physiotherapists discussed the changing nature of disease that necessitates continual monitoring to respond to changes and review them.

“If you choose the right cue and you review it—because some people, as cognition changes or a stress has become worse for them, their focus or their ability to have previously managed a cognitive task for the cueing is—becomes more difficult and you need more external cues than internally-driven movement strategies. (Participant 7)”

#### Subtheme 2: building a trusting relationship

Physiotherapists perceived effective communication with PwP as a means to optimize compensation strategies. They shared that training compensation strategies is a lengthy process that requires establishing trust.

“We have support workers and often we will ask them to go maybe two or three times a week to reinforce it. So, they can […] help sort of build it into a strategy more longer-term as opposed to doing it with the physio once. (Participant 10)”

This was evident in physiotherapists' accounts of their interactions with PwP and highlighted the importance of building relationships. For example, the longer follow-up enabled them to build better relationships with patients to gain their trust and confidence. Thus, communication was a bridge that enabled them to tailor these strategies.

Physiotherapists were aware of PwPs' motivations to use strategies and how this could underpin their beliefs about the benefits. They noted how patients' commitment to using strategies could be driven by motivation.

“Their belief in whether it's going to work or not. Some of them will really try and practice it and want to keep practicing it with you, and some of them will sort of half-heartedly have a go and then think it's not for me and it doesn’t work. (Participant 8)”

Physiotherapists discussed how meaningful engagement could enhance PwPs’ motivation to use strategies. For example, recording videos to demonstrate walking progress could raise patients' awareness of the benefits of the strategies. Thus, it was viewed as a means of gaining patients' trust and, eventually, motivation.

“Trying to explain, on the right level, why we’re trying to do the things that we’re doing and then hopefully it, kind of, then embeds … I am not just randomly telling you to step over lines for absolutely no reason; there is some logic to asking you to do it, and I think, hopefully, that you know it helps with therapy to try and say, you know, this is why we’re doing what we’re doing and hopefully to make some them more motivated and, you know, more aware (Participant 11).”

The language physiotherapists used was another clear way they engaged with PwP. For example, they enhanced the PwP's understanding of the impact of the disease on their body through education about strategies, using appropriate language such as analogies, which improved the PwP's motivation to use compensation strategies.

“The idea is to try and get these bigger movements … I always describe it to my patients—like the internal short-circuit that goes on that limits their movement patterns [It is important] to try and overcome this by giving them a focus-targeted approach to overcome that short-circuiting. (Participant 8)”

#### Subtheme 3: home-based involvement

Physiotherapists considered enabling PwP's use of compensation strategies in their environment. This is because the difference between the clinical environment and home can impact the patient's ability to manage the situation. As a result, physiotherapists modified compensation strategies according to the available resources that would enable PwP to adopt strategies.

“It has to be something that they can do outside of the physio session. So, it's finding something that is usable outside of that clinical setting and that period of time. So, the metronome was questionably useful. (Participant 10)”

Although physiotherapists strove to enable PwP to independently use compensation strategies, in some cases, involving family members was needed to enhance the strategies' effectiveness. This was particularly relevant if the patient had cognitive problems that affected their ability to perform the strategies daily when support was needed. For example, some PwP required help, supervision, and reminders to adopt compensation strategies, but that relied on whether the family was willing to be involved in the management. Physiotherapists reported that the patients did not always have a relationship with their caregiver or spouse or a supportive family. They referred to the impact that Parkinson's has on the family and discussed how it could be an extra burden for families that already have other responsibilities.

“There's something about trying to preserve their relationship a little bit and not expecting that carer or that partner to be burdened by it unless they’re well up for it. (Participant 13)”

#### Theme 2: delivering compensation strategies effectively

Although the personalized approach was vital to optimize compensation strategies in PwP, physiotherapists described several challenges and support in relation to 1) work settings and 2) experience that impacted their ability to deliver compensation strategies effectively.

#### Subtheme 1: work settings

The rehabilitation goals within work settings appeared to shape physiotherapists' ability to maintain person-centered care for PwP related to their rehabilitation potential, especially regarding gaining specific information about the nature of the patient's Parkinson's disease. For example, physiotherapists working in medical wards of both acute and subacute hospitals discussed the time constraints they faced as they tried to gain specific Parkinson's knowledge in addition to knowledge of the other conditions they encountered daily. A lack of resources compounds this: the focus of the care is not always specifically neuro-rehabilitation. In contrast, physiotherapists working in rehabilitation wards (e.g., community neurorehabilitation) were able to deliver a more personalized approach to PwP because of the support available within their work settings. For example, they worked with neurology teams to individualize rehabilitation plans. Furthermore, they received in-service training.

“Mainly because the people that have been delivering the training for us are neuro-specialists … so, they’re easily accessible to me … when we’ve attended external courses in the past, it's very difficult sometimes to get access again to the tutor. Whereas I’ve got quite good access and quite good, sort of, working relationships with my colleagues in the neuro team. So I can speak to them afterward. I can ask them questions. I can, you know, problem-solve individual cases—that sort of thing. So, it's been really helpful. (Participant 4)”

Physiotherapists in the community and private sectors discussed how longer follow-ups enabled them the time to build relationships with patients that are not possible when working in a hospital setting, when input is often very short-term. This enabled them to respond to changing patient needs, through tailored approaches to ongoing monitoring and input. For example, they could follow up in the clinic, at home or using technology, enabling them to constantly review patients to ensure they were adopting compensation strategies effectively.

 “Most of us as physiotherapists have the ability to go to someone's house. Most of us can do a home visit. If we can’t—just say you’re in an NHS ward-base, and you can’t do that home visit—[we] set it up so that the community physio can. Working in the private sector allows me the choice of going to someone's home or staying in the clinic. And sometimes, if there's a spouse available, or if the person with Parkinson's can set it up, just carry out the appointment using digital technology. Most people are on Zoom or FaceTime. (Participant 7)”

#### Subtheme 2: evidence, experience, and expertise

A profound challenge for physiotherapists was the lack of formal training in compensation strategies; instead, most of the physiotherapists' experience was gained from learning from their peers. Although physiotherapists reported formal training in other interventions, such as PD Warrior programme (25), no specific training courses were available for using compensation strategies. This shortcoming had many implications for their practice. For example, understanding the mechanisms underpinning each strategy's potential efficacy impacted their confidence in providing patient education.

“It's understanding how, like a cueing situation, how that exactly is processed in your brain so that I’ve got the confidence and the education … so that I give it in layman's terms, but I think [I] need a bit more clarity myself. So, by saying something and by doing a movement, how does that trigger something in the brain of someone with Parkinson's? I’ve never had formal education of that. (Participant 6)”

Specialized physiotherapists also emphasized the need for Parkinson's-specific education:

“In terms of giving the information … I think it helps to understand the basal ganglia and how they function normally and then how they are dysfunctional in Parkinson's because that helps you understand why the movement strategies work. (Participant 5)”

There were different perspectives on the focus of training/education on compensation strategies. Some physiotherapists discussed the importance of training/education to enable the effective delivery of compensation strategies, given the complexity of Parkinson's, as these strategies work differently in PwP. Others pointed out that such knowledge is not enough, and clinical experience is also needed to deliver them effectively.

“A lot of the literature is very much giving you the theoretical principles as to how cueing would work, but [It] doesn’t necessarily tell you how to prescribe it. And, also, I think, probably, from a personal point of view, how you prescribe cues for one person will be quite different from how you prescribe cues to another person. So, I don’t necessarily think there's an awful lot of guidance or a framework to work through about how to apply cues … one of the challenges for [physiotherapists] is that there are very few Parkinson's-specific specialist [physiotherapists]. And that's what I think. I think people need to have an understanding of how to adapt these interventions to meet the hugely diverse needs of people with Parkinson's. (Participant 9)”

Physiotherapists also suggested that both knowledge and clinical application were needed.

“It's a two-way relationship, really … In the research world, we really need to get to the bottom of things, but you need to have the experience, I think, clinically, to know why you’re asking the questions. I think, especially with things like cues, when you do them clinically, you know, with some people it works, some people it doesn’t, and we just don’t know why. (Participant 11)”

## Discussion

This study explored physiotherapists' experiences using compensation strategies to improve walking in PwP. In this study, physiotherapists often personalized their practice to optimize compensation strategies. Their approaches were driven by their awareness of the need to tailor compensation strategies according to PwP characteristics, needs, and preferences. This holistic approach was justified by the dynamic nature of Parkinson's disease and patients' needs, which necessitate continual monitoring to ensure these strategies are effective and flexible over time. Although personalized care was vital to optimizing compensation strategies in PwP, other factors impacted physiotherapists' ability to deliver these strategies effectively.

“Person-centeredness’ refers to a philosophy intended to underpin care and service delivery focused on: meeting the person's needs, values or preferences; optimizing the person's experiences with care; and fully involving persons’ perspectives into care (26).” Physiotherapists' approaches to optimizing compensation strategies are congruent with the evidence from previous research on the personalized approach to gait rehabilitation in PwP (11). Although physiotherapists strove to optimize compensation strategies, there was a lack of formal training on this subject, and physiotherapists' knowledge was primarily acquired from their peers. This may draw attention to the lack of the necessary knowledge that enables physiotherapists to be person-centered. A systematic review of person-centered physiotherapy reported that physiotherapists should be confident and show the patients through communication that they have the required knowledge (27).

In our study, Parkinson's specialist physiotherapists draw on theoretical aspects (e.g., the loss of automatic control) to provide an appropriate education. They also emphasized building a trusting relationship and promoting their motivation through meaningful engagement to use compensation strategies. However, some physiotherapists reported that a lack of knowledge affected their confidence in providing this education. This has implication for goal setting toward personalized approach. Thus, the lack of specific knowledge on Parkinson's can impact physiotherapists’ confidence in maintaining person-centered rehabilitation. Furthermore, physiotherapists working in medical wards highlighted the time constraint to develop such specific Parkinson's knowledge. This agrees with the findings of other studies, in which contextual factors such as workplace environment have been shown to influence physiotherapists' decision-making process in clinical practice (28). Future research should establish a theoretical multifactorial framework to promote the effective prescription of compensatory strategies in PwP that includes training in how compensation strategies work and how to prescribe and tailor strategies, whilst ensuring adequate time to understand the PwP changing and fluctuating needs and to enable monitoring.

Physiotherapists in the current study discussed managing the non-motor symptoms to personalized compensation strategies as well as the need to refer to another role of the multidisciplinary rehabilitation team. This is especially the case managing the freezing of gait that is caused by the anxiety of walking. Prior studies discussed the collaboration between physiotherapists and occupational therapists in adapting the environment to the patient using walking aids (29). However, little is known about this collaboration in managing the freezing of gait caused by the anxiety of walking. Skelly and colleagues support this and emphasize the multidisciplinary approach for PwP due to the physical and psychological characteristics of Parkinson's disease (30). However, non-motor symptoms (e.g., the cognitive deficit) and their impact on walking are not fully understood.

Part of the physiotherapists' approach to optimizing compensation strategies was addressing the cognitive deficit of Parkinson's disease and its impact on walking (e.g., anxiety around freezing) and referring to other roles in the multidisciplinary rehabilitation team. A narrative review by Radder and colleagues discussed the overlapping between physiotherapists and occupational therapists and suggest using the International Classification of Functioning, Disability, and Health (29). They highlighted managing freezing by adapting to the environment around the patient and using walking aids. Our study contributes to understanding the collaboration between these disciplines in managing freezing in the cognitive elements and their interference with walking. In everyday practice, physiotherapists should identify whether a patient's non-motor symptoms are related to capacity and performance or are a symptom that requires help from another health professional.

Previous research has emphasized the importance of maintaining ongoing conversations between multidisciplinary team members due to the complex nature of Parkinson's and the changes in patient needs over time (31), especially due to the importance of multidisciplinary collaboration to improve patient outcomes (32). This has important implications for personalized care for PwP and understanding the collaboration required between disciplines to manage non-motor symptoms that affect walking in PwP. This may agree with a survey using “multidisciplinary” perspectives (including other healthcare practitioners such as occupational therapists and psychologists); despite the study's conclusion about the inconsistency of explicit and implicit learning, the survey emphasized the distributed expert opinions regarding applying motor learning to clinical practice (9). Thus, this may suggest that part of the personalized approach is understanding the multidisciplinary team roles of training such intervention. Further work is required to establish physiotherapists’ roles within the multidisciplinary team to enhance gait rehabilitation for PwP.

The study findings suggest that given physiotherapists' lack of formal training regarding compensation strategies for PwP, developing such education may be indicated. In particular, integrating Parkinson's-specific knowledge reported by specialist physiotherapists may help deliver personalized care and optimize compensation strategies effectively. However, a question remains on the nature of accessible training that could address the knowledge–practice gap to contribute to the delivery of better-personalized care for PwP. While it has been proposed that compensation strategies may promote motor learning (33), it is difficult to translate existing motor learning theory into physiotherapy practice (34). For instance, one phenomenological study aimed to understand physiotherapists' perceptions of motor learning–based practice and reported: “complexity in the field and the lack of clarity regarding its theoretical content and clinical applications” (35). Recently, Leech et al. described several mechanisms of motor learning that contribute to developing evidence-based practices (36). However, this mechanism is poorly understood in PwP, most likely due to the heterogeneity of the condition and anecdotal observations that every patient is different and demonstrates their unique response to a given compensation strategy.

In addition to what has previously been mentioned, different compensation strategies work differently for everyone. Thus, given the scarcity of literature on how motor learning theory should be applied, it is challenging to use it to inform physiotherapist training. Therefore, these considerations of physiotherapists' access to specific Parkinson's knowledge and the training that informs their practice need to be addressed in future research.

## Strengths and limitations

A number of limitations need to be noted regarding the present study. First, regarding the number of participants, the sample size is controversial in qualitative research (37). By adopting the model of information power over data saturation (19), our study used rich interview data from 13 physiotherapists with different ranges of Parkinson's expertise and work settings around the UK to answer the research questions. Second, although the recruitment was not specifically designed to focus on females, most participants were female. While this is likely a reflection of the people who volunteered to participate in the study, it might be because the majority of physiotherapists in the UK are female (38). Furthermore, most of the study participants were employed by NHS, the main healthcare provider in the United Kingdom. Thus, these findings is limited to the UK context and may not be transferable to other locations.

## Conclusion

The study provides insight into physiotherapists' approach to optimizing and delivering compensation strategies in PwP. Although physiotherapists strived to optimize compensation strategies, there was a lack of formal training in this area, and their knowledge was primarily acquired from peers. Furthermore, a lack of specific knowledge on Parkinson's can impact physiotherapists’ confidence in maintaining person-centered rehabilitation. However, the question that remains to be answered is what accessible training could address the knowledge–practice gap to contribute to the delivery of better-personalized care for people with Parkinson's.

These findings add to a growing body of literature that indicates compensation strategies work differently in PwP, and to optimize them, they need to be individually tailored to ensure that they work for the patient specifically. This study suggests developing a theoretical multifactorial framework to promote the effective prescription of compensatory strategies in PwP. This should include how compensation strategies work and how to prescribe and tailor strategies.

Future research should focus on determining the training needs to empower physiotherapists to deliver person-centered practices. Further work is required to establish physiotherapists' roles within the multidisciplinary team of training compensation strategies to enhance gait rehabilitation for PwP.

## Data Availability

The datasets presented in this article are not readily available to protect confidentiality. Requests to access the datasets should be directed to v.goodwin@exeter.ac.uk.
