# Supplementary material for: “It's a lot more complicated than it seems”: physiotherapists' experiences of using compensation strategies in people with Parkinson's
Source: Front Rehabil Sci. 2023 Jun 5;4:1157253. doi: 10.3389/fresc.2023.1157253 (PMC10277694; doi:10.3389/fresc.2023.1157253)
Supplement: Supplementary file 1 [file Datasheet1.docx]

**Interview Topic Guide**

**How do physiotherapists learn about using movement strategies?**

- Can you introduce yourself and tell me briefly about how your role involves working with people with Parkinson’s disease?
- Have you received any training in using movement strategies? If yes, do you find it helpful?

**How and why do physiotherapists use movement strategies?**

- When you use these strategies, how do you decide which to use? [Prompt: Can you give any examples?].
- Was there anything you took into consideration when you decided to use these strategies? [Prompt for training, reading, peer influence, prior experiences, and perceived patient acceptability].
- Explore how movement strategies may differ between patients, such as patients’ characteristics (Parkinson’s severity, cognitive decline) and individual differences.

**The effectiveness of movement strategies**

- What changes in patients are you looking to achieve as a result of particular strategies?
  - [Prompt for motor control and movement, presence of motor and non-motor symptoms, whether different strategies result in different outcomes, and provide examples].
- If a certain strategy may not have worked, can you tell me about other strategies you then used?
- In what circumstances do you think the effect of a particular strategy may differ?

**How do physiotherapists assess the effectiveness of strategies?**

- How do you think movement strategies may benefit patients?
- Do you think about anything that may affect patients’ responses to these strategies?
  - [Prompt for the nature of the patient’s stage of Parkinson’s and their family, social, and cultural context].
- Explore patients’ motivation to use movement strategies.
- Is there anything you would like to add?
